# The Effect of Salbutamol and Budesonide Pediatric Doses on Dental Enamel and Packable and Flowable Composites: Microhardness, Surface Roughness and Color

**DOI:** 10.3390/pharmaceutics15112527

**Published:** 2023-10-25

**Authors:** Maria Salem Ibrahim, Fatimah Mohammed Alatiyyah, Khawla Abbas Mohammed, Hibah Nouh Alhawaj, Abdulrahman A. Balhaddad, Ahmed Salem Ibrahim

**Affiliations:** 1 Department of Preventive Dental Sciences, College of Dentistry, Imam Abdulrahman Bin Faisal University, Dammam 34212, Saudi Arabia; 2 College of Dentistry, Imam Abdulrahman Bin Faisal University, Dammam 31441, Saudi Arabia; 2180006396@iau.edu.sa (F.M.A.); 2190004503@iau.edu.sa (K.A.M.); 2190005342@iau.edu.sa (H.N.A.); 3 Department of Restorative Dental Sciences, College of Dentistry, Imam Abdulrahman Bin Faisal University, Dammam 31441, Saudi Arabia; abalhaddad@iau.edu.sa; 4 Medical Microbiology Department, Health Monitoring Centers, Ministry of Health, Jeddah 21176, Saudi Arabia; ahsibrahim@moh.gov.sa

**Keywords:** restorative materials, anti-asthmatic, tooth brushing, composite, enamel, pediatric

## Abstract

Objective: To assess and compare the effects of two pediatric anti-asthmatic medication doses on the microhardness of enamel and microhardness, surface roughness and color of restorative materials. Methods: Human enamel samples and packable and flowable composite restorations were used. The samples were exposed to Salbutamol (0.6 mL/6 mL saline) and Budesonide (2 mL/2 mL saline) via a custom-made chamber connected to a nebulizer. Medication administration was conducted for 10 days. The samples were brushed with an electronic brush in a continuous and circular mode for 10 s after 10 min of medication administration. Assessments of microhardness, surface roughness and color were carried out at three different time intervals: baseline (T_0_), 5 days (T_1_) and 10 days (T_2_). One-way analysis of variance (ANOVA), a two-sample t-test and a Bonferroni multiple comparison test were used to analyze the data and compare between the groups. Results: Both medications significantly (*p* < 0.05) decreased the microhardness of the enamel and composite samples after 10 days. Both medications lowered the surface roughness of both types of composite with a greater effect observed after 10 days of Budesonide administration (*p* < 0.05). Both medications had comparable detectable color change on both types of composite with a greater effect observed after 10 days of Budesonide administration (*p* < 0.05). Conclusion: Salbutamol and Budesonide significantly decreased microhardness in the enamel samples. Both medications affected the properties of packable and flowable composites. The packable composite showed more resistance to microhardness changes. Both medications showed a clinically detectable change in the color of packable and flowable composites.

## 1. Introduction

Asthma is a disease with persisted inflammation of the lower airways that affects people of all ages. It is characterized by variable airway hypersensitivity, airflow obstruction and symptoms like wheezing, chest tightness, breathlessness and coughing [1]. It has an adverse effect on patients, families and communities [1,2]. Genetics has been shown to be a predisposition factor. Environmental interactions with genetic and host factors lead to the high prevalence of asthma [3,4]. According to the World Health Organization (WHO), an estimated 235 million, both children and adults, worldwide have asthma. Asthma is among the top 20 illnesses in the world for children. Globally, 0–0.7 deaths per 100,000 persons occur due to asthma in children [5,6]. Studies have shown that the oral cavity may be affected by chronic asthma treatments via inhaled treatment medications [7,8]. It was shown that bronchial asthma has a detrimental effect on the oral cavity by reducing salivary secretion and changing saliva composition and pH [3,5]. Furthermore, low pH can lead to demineralization of the tooth structure and may lead to caries formation. After 30 min of using an inhaled medication, there was a significant reduction in the salivary pH to below the critical value of 5.5 for enamel demineralization [3,9]. Anti-asthmatic medications and inhaled corticosteroids are associated with xerostomia, dental caries, gingivitis and periodontitis as well as oral mucosal changes like candidiasis [10,11,12].

There are different types of inhaled drugs used as a treatment for asthma such as beta-2 agonists, anticholinergic bronchodilators, inhaled corticosteroids and sodium cromoglycate. They can be used in a combination or alone [11,13]. Treatments used in inhaled form show some negative impact on oral health. The impact varies depending on the dosage, duration and frequency of use. High dosage and long duration of inhalation treatment have been linked with several negative effects on the oral cavity. A major proportion of the inhaled drug is retained in the oral cavity and oropharynx so it may interfere with the normal physiology of oral tissues [10,12].

Salbutamol is one of the common anti-asthmatic inhalers. It is a beta-2 agonist fast-onset bronchodilator drug that is used in routine management of chronic bronchospasm in all ages [4,14]. Several studies showed an important role of Salbutamol in oral health and smooth muscles as its prolonged use might change the oral environment leading to an increase in caries risk and causing gastro-esophageal reflux [6,14]. Another type of inhaler used to treat asthmatic inflammation is Budesonide, which is a corticosteroid [7,15]. It was developed to address the needs of newborns and young children with persistent asthma [15]. The only inhaled corticosteroid authorized in the United States to be used with children as young as 12 months old is Budesonide [15,16,17]. This medication was also the first corticosteroid licensed to be used with a nebulizer [15]. Budesonide has a potent anti-inflammatory effect in asthma, chronic obstructive pulmonary disease and other respiratory illnesses [15,17].

However, there are few studies that have compared the effect of these inhalers after brushing on color and surface roughness of dental composites and microhardness of dental composites and enamel surface. This study aimed to assess the effects of Salbutamol and Budesonide administration for 5 days and 10 days on the surface roughness, color and microhardness of packable and flowable composite and microhardness of teeth. The null hypotheses stated that the anti-asthmatic medications would not have a significant effect on the microhardness of enamel structure nor on the surface properties of both composite types.

## 2. Materials and Methods

### 2.1. Study Design and Ethical Approval

This study was designed as an in vitro laboratory study. Approval was obtained from the Institutional Review Board at Imam Abdulrahman bin Faisal University (IRB-2022-02-439).

### 2.2. Sample Size

The sample size for teeth samples was calculated based on data from a previous study that assessed surface hardness after an inhaler exposure (316 ± 41.6) in comparison with a control group (270 ± 37.2) with α = 0.05 and power = 80% [9]. The calculated sample size was 13 samples per group.

The sample size for composite samples was calculated based on data from a previous study that assessed surface roughness after an inhaler exposure (5.43 ± 1.16) in comparison to a control group (2.63 ± 0.82) with α = 0.05 and power = 80% [18]. The calculated sample size was eight samples per group.

### 2.3. Sample Preparation

#### 2.3.1. Teeth Sample Preparation

Extracted human teeth were collected and stored in 0.01% (*w*/*v*) thymol solution (pH 7.0) at 4 °C until use. Teeth were sectioned to produce enamel blocks measuring 3 by 3 mm and 2 mm in thickness using diamond discs. The samples were then fixed in an acrylic resin that was fabricated using a circular mold [9,12,19]. Samples were polished using a grinder polisher with a vector power machine (EcoMet™ 30 Semi-Automatic Grinder Polisher, Buehler, IL, USA) using abrasive discs with 320, 600 and 1200 grit silicon under water coolant. The final polished surfaces had uniform orientation and were free of cracks, deformations, steps and slopes.

#### 2.3.2. Composite Sample Preparation

Disc-shaped samples (10 mm in diameter and 2 mm in thickness) of Nanohybrid packable resin composite (Tetric^®^ N-Ceram, TBF, Ivoclar/Vivadent, Schaan, Liechtenstein) and Nanofilled flowable composite (Tetric^®^ N-Flow, TBF, Ivoclar/Vivadent, Schaan, Liechtenstein) were prepared (Table 1). Each material was injected into a mold which was covered with clear polyester strips and glass slides from both sides and then light-cured for 20 s on each side (Satelec Mini LED Curing Light 1250 mW/cm^2^, A-dec Inc., Newberg, OR, USA) following the manufacturer’s instructions. The sample edges were polished with sandpaper after 24 h [20,21,22]. The samples were then divided randomly between the two groups of medications (*n* = 8).

### 2.4. Medication Administration and Brushing Technique

**Figure 1:**
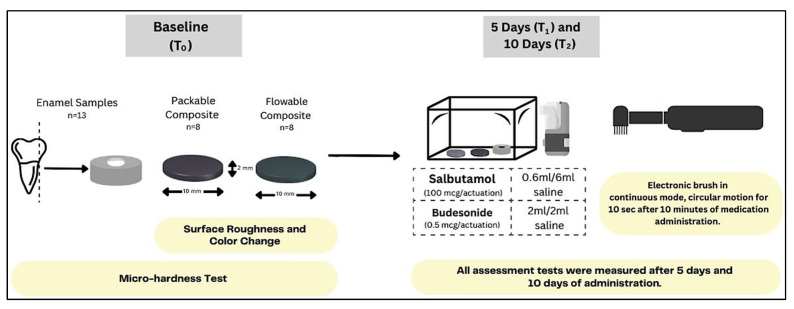
Schematic drawing of the study flow.

The samples were put inside a custom-made chamber which was fabricated to be sealed and represent the patient’s mouth during medication administration. A special hole was designed to allow the opening tube of an ultrasonic inhaler mesh nebulizer to spread the material inside the box and mimic the clinical scenario. Samples were exposed to Salbutamol or Budesonide mixed with saline as mentioned in Table 2 based on the recommended doses. During the medication administration, the samples were fixed inside the box to expose their surfaces. To test the brushing effect, the tested surfaces were brushed with an electronic brush in a continuous and circular mode by one operator who brushed each sample for 10 s after every single dose as shown in Figure 1 [20]. This duration was calculated with the consideration that each tooth would be brushed for 10 s. For the standardization of medication doses, each sample was given the maximum daily doses for pediatric patients. Administrations of the medications were carried out once daily for 10 days.

### 2.5. Assessments

The testing assessments were measured before the application (T_0_), after 5 days of the medication administration (T_1_) and after 10 days of administration (T_2_).

#### 2.5.1. Microhardness

##### Teeth Samples

A Knoop hardness test was conducted using a hardness tester (BUEHLER MicroMet 6040 Hardness Tester, Shanghai, China). Five indentations at 25 g and 10 s dwell time were made per sample, and the average of these readings was taken to have the final hardness value for that sample [9,12,19]. Samples were included in the study with an average microhardness of 430 KH ± 20%, and then they were randomly and blindly distributed among the groups. The test was carried out at T_0_, T_1_ and T_2_ for each sample.

##### Restorative Composite Samples

A Vickers hardness test was performed using a hardness tester (BUEHLER MicroMet 6040 Hardness Tester, Shanghai, China). Five indentations at 200 g and 10 s dwell time were made per sample, and the average of these readings represented the final hardness value for that sample [23]. At T_0_, the average microhardness was 100.6 ± 12 VH for packable composite and 76 ± 10.9 VH for flowable composite. Then they were randomly distributed among the groups. This was repeated at T_1_ and T_2_ for each sample.

#### 2.5.2. Surface Roughness

A surface roughness measurement was measured using a noncontact profilometer (Contour GT-K 3D Optical Microscope, Bruker, Billerica, MA, USA) [24]. Samples were dried using absorbent paper and evaluated for surface roughness (Ra, nm) at three areas: the center and two points apart from it. This was repeated at T_0_, T_1_ and T_2_ for each sample.

#### 2.5.3. Color

A reflectance spectrophotometer (Color-Eye 7000A; X-rite, Grand Rapids, MI, USA) was used to measure color change. The Commission Internationale de l’Elcairage (CIE) L*a*b*color scale was used to measure the color of the tested samples at T_0_, T_1_ and T_2_. The CIE L*a*b* system represents a three-dimensional color space with L* representing lightness from black to white on a scale of zero to 100, while a* and b* represent chromaticity with no specific numeric limits. Negative a* corresponds with green, positive a* corresponds with red, negative b* corresponds with blue and positive b* corresponds with yellow [25]. The three-color coordinates were recorded at the three different time intervals, and ΔE* was calculated (between baseline and different administration durations) using the formula [20,24]:

ΔE* = [(ΔL*)^2^ + (Δa*)^2^ + (Δb*)^2^]^1/2^

The equation was used to measure ΔE at T_1_ and T_2_.

At T_1_,

ΔL* = L_1_ − L_0_, Δa* = a_1_ − a_0_ and Δb* = b_1_ − b_0_

At T_2_,

ΔL* = L_2_ − L_0_, Δa* = a_2_ − a_0_ and Δb* = b_2_ − b_0_

L_0_, a_0_ and b_0_ were the initial measured color data; L_1_, a_1_ and b_1_ were the measured color data after 5 days of medication administration; and L_2_, a_2_ and b_2_ were the measured color data after 10 days of medication administration.

### 2.6. Statistical Analysis

The Shapiro–Wilk test was used to check the data normality before analysis. The results of microhardness, surface roughness and color change were presented by means and standard deviations and then analyzed using one-way analysis of variance (ANOVA) and/or a two-sample t-test. Multiple comparisons between the different groups were conducted using the Bonferroni multiple comparison test. The statistical analyses were performed by Stata/IC 14.2 (Stata, College Station, TX, USA). GraphPad Prism Version 9.5.1 (GraphPad Software, Boston, MA, USA) was used to create the graphs.

## 3. Results

**Figure 2:**
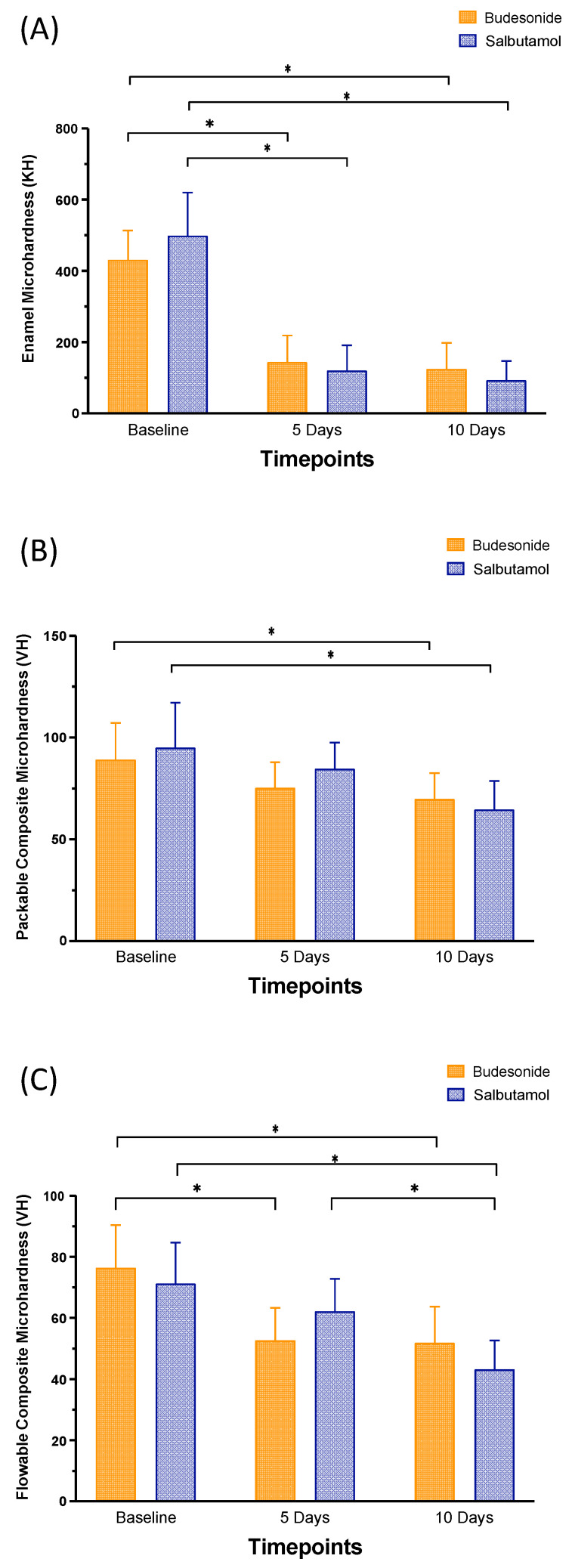
Microhardness (KH or VH) values (mean ± SD) of the studied groups: enamel (**A**), packable composite (**B**) and flowable composite (**C**) at different time intervals: baseline (T_0_), 5 days (T_1_) and 10 days (T_2_) after brushing after Budesonide (orange) and Salbutamol (blue) administration. Stars denote statistically significant difference between the groups (*p* < 0.05).

Figure 2 and Table 3 summarize the changes in the microhardness of the enamel, packable composite and flowable composite samples after 5 days and 10 days of medication administration and brushing. Both medications significantly (*p* < 0.05) decreased the microhardness of the enamel samples after 5 and 10 days of each medication administration. There were significant reductions in the microhardness of packable composite after 10 days of medication administration (*p* < 0.05). Both medications significantly (*p* < 0.05) decreased the microhardness of the flowable composite samples after 10 days of each medication administration.

**Figure 3:**
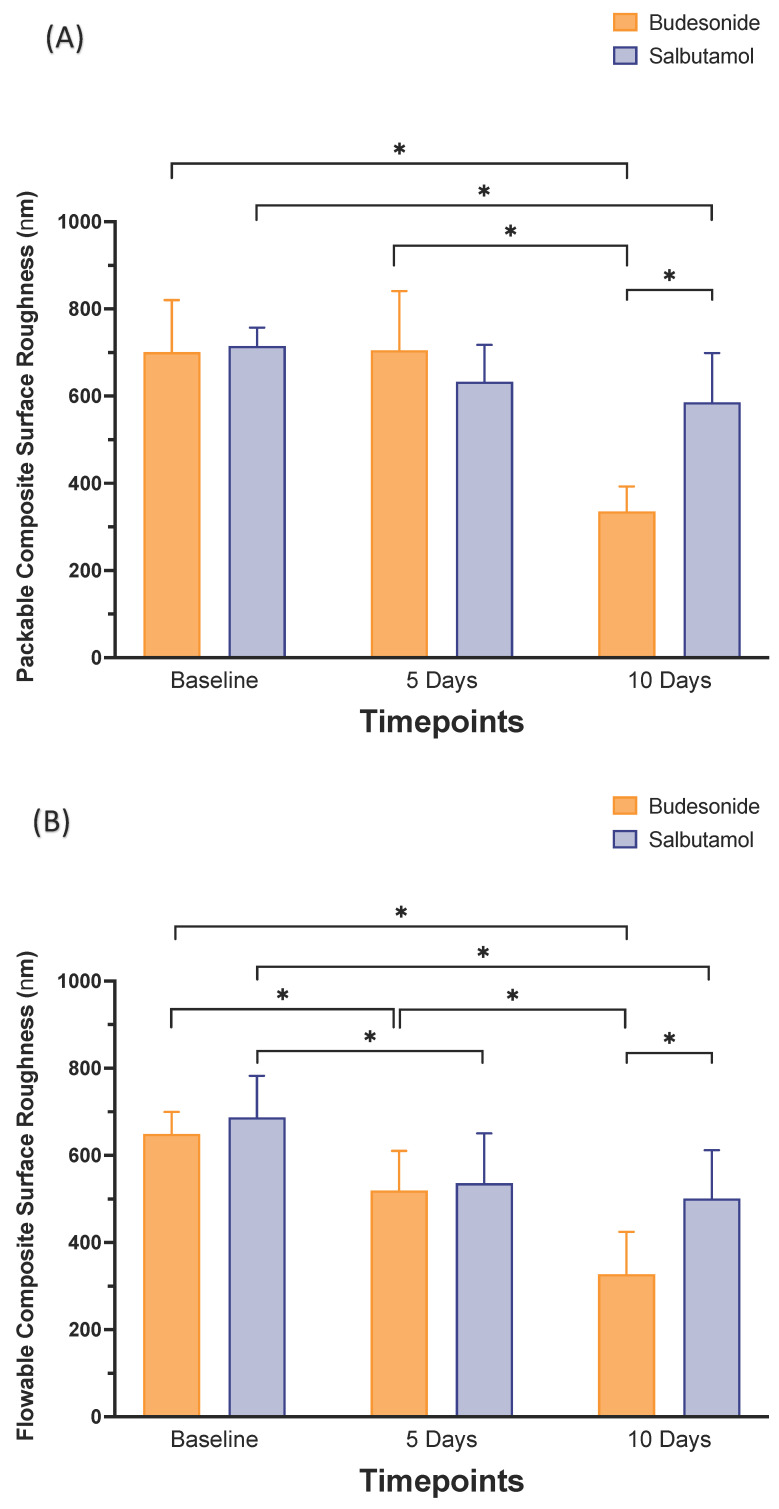
Surface roughness (Ra) in nm (mean ± SD) of the studied groups: packable composite (**A**) and flowable composite (**B**) at different time intervals: baseline (T_0_), 5 days (T_1_) and 10 days (T_2_) after brushing after Budesonide (orange) and Salbutamol (blue) administration. Stars denote statistically significant difference between the groups (*p* < 0.05).

Both medications decreased the surface roughness of both packable and flowable composites (*p* < 0.05) after 10 days of each medication administration and brushing. Budesonide showed a higher reduction (*p* < 0.05) in surface roughness of both packable and flowable composites after 10 days of application (Figure 3 and Table 4).

**Figure 4:**
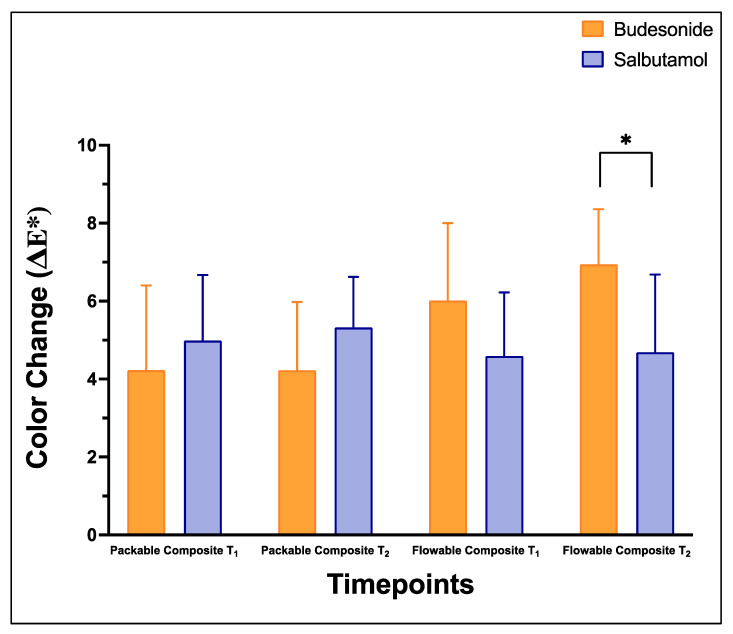
Color change (ΔE*) values (mean ± SD) of the studied groups: packable composite and flowable composite at different time intervals: baseline (T_0_), 5 days (T_1_) and 10 days (T_2_) after brushing after Budesonide (orange) and Salbutamol (blue) administration. Stars denote statistically significant difference between the groups (*p* < 0.05).

Figure 4 and Table 5 demonstrate the means and SDs of color changes (ΔE*) following medication administration and brushing at T_1_ and T_2_. Both medications, Salbutamol and Budesonide, had comparable effects on packable composite in terms of color change values at T_1_ and T_2_ (*p* > 0.05). The flowable composite at T_2_ showed significant lower color change after Salbutamol administration than after Budesonide administration (*p* < 0.05).

## 4. Discussion

Anti-asthmatic medications are needed to help in controlling asthma. These medications may affect the oral health of pediatric, adolescent and adult patients. Therefore, oral hygiene has a major role in achieving good oral health while using medications [3,8,11]. One of the major effects of these medications is having low pH of 3.6–4. This pH was found to have an erosive effect that promotes enamel dissolution and material and resin degradation over time [9,25,26,27]. In clinical practice, oral liquid medications and aerosols are typically prescribed for pediatric patients for frequent chronic use. However, these medications contain additive components such as sucrose, fructose and glucose. This can initiate the bacterial fermentation cycle by producing acids and lowering the pH of the oral environment, leading to increased risk of degradation of hydroxyapatite crystals [9,12,28].

In this investigation, investigators assessed the change in microhardness, surface roughness and color after medication administration and brushing for 10 days. Samples of teeth and restorative materials were exposed to one of two of the most used anti-asthmatic medications, Salbutamol and Budesonide. The medications were applied using a nebulizer attached to an acrylic box to mimic the actual method of application. After application, samples were brushed using an electric brush to assess the impact of medication absorption by the samples. These medications and compounds may leave a deposit after inhalation and coat the teeth and periodontal tissues while being inhaled [10]. Therefore, it is important to assess the effect of brushing after medication application while the residual components are coating the teeth [26,29].

Previous studies have tested the immersion of enamel blocks in anti-asthmatic medications. They found a significant decrease in enamel surface microhardness after immersion in inhaler solutions [9,12,30]. They found that both Salbutamol and Budesonide inhalers at 5 and 10 days of use had decreased the enamel surface microhardness with the Budesonide-based inhalers having the highest erosive effect [9]. The immersion in the solutions may increase the breakdown due to the constant contact with the solution. In this study, samples were exposed to the medications using a nebulizer rather than immersion in the medication solutions to resemble the actual clinical scenario. Both medications had reduced the enamel samples’ microhardness as well as that of the packable and flowable composites. In previous studies, they found a significant reduction in microhardness following the administrations of both medications on enamel and different restorative materials [9,18]. Also, flowable composite showed a higher reduction in microhardness in comparison to the packable composite after 5 days. When exposed to acidic conditions, composite resin rapidly releases monomers leasing to microcracking. This may raise the absorption and retention into the resin matrix and lead to a softer resin matrix that worsens the damage as the separation between polymer chains gets broader [26,27]. The hydrolysis of silane-binding agents and the breakdown of chemical bonds between the filler particles caused by water absorption can accelerate the degradation of dental restorations [27,31,32]. This could account for the finding that the packable nanohybrid composites in the current investigation had produced superior overall outcomes than the flowable composite.

An important clinical property for any restorative material is its surface roughness. More plaque retention, bacterial adhesion and gingival irritability may result from increased roughness [33,34]. For dental materials, surface roughness and color characteristics are connected since restorations with high surface roughness are more likely to stain [21,24]. The color of the restoration also depends on the surface roughness because a smooth surface reflects lighter color than a rough surface [18,24]. In this study, medications showed a beneficial effect by reducing the surface roughness of composite resin restorations in contrast to previous studies [21,24]. This could be justified by diluting the medications in saline alongside the brushing action [29,35]. At the end of 5 days of both medication administrations, the flowable composite showed a higher reduction in surface roughness whereas the packable composite did not show any significant changes. However, after 10 days, the surface had a smoother texture in both types of restoration. Surface roughness is directly influenced by the organic matrix structure and filler properties. The behavior of the resin matrix and the type and quantity of fillers in composite resins are the major causes of composite failure [27,35]. This may justify the fact that the flowable composite was affected by both medications faster in comparison to the packable composite.

In this study, both types of composite showed a clinically undesirable color change following the medication administrations with no significant differences between the two types or two medications or time intervals except in between the two medications after 10 days in the flowable composite groups. Color change that the observers could detect by visualization was set as ΔE > 3.3 [25,36]. The results supported previous studies that revealed that medication inhalation had an impact on color stability of dental restorations [20,21,24,37]. The components of any medication, pigments present in medications and exposure time are some of the factors that may affect the color stability of any restorative material. The sulfate group that is included in the inhaler nebulas is an active component which attaches to the surface and enhances adherence and discoloration [20,32]. Administration of the lactose monohydrate-containing dry-powder form of Salbutamol can reduce salivary flow, raise bacterial counts and lower the pH, which increase the incidence of caries in asthmatic patients [16,28,32]. Thus, brushing with different oral hygiene measures is fundamental and should be reinforced for asthmatic patients. Brushing the teeth improved the color stability of the restorative materials [23]. When previous studies examined the effects of colored drinks on a variety of restorative materials used in primary teeth, they found that the brushed samples had less color change than the other subgroups that did not have the brushing factor [29,35,38]. Although the brushing action still did not completely eliminate the color change after medication administration, the level of color change was just above the clinically detachable level. This could reflect the importance of brushing to minimize the effect of different medications on the color of different composites. In addition to the brushing action in this study, having saline mixed with the medications may help in reducing the effect on the color and in reducing the surface roughness as found. By adding saline, the viscosity of the medications would be reduced, and their adherence to different surfaces would be decreased. In contrast, the higher the viscosity of the medications, the higher their ability to adhere to the tooth’s surface for a longer period [11]. In clinical settings, the found undesirable color change in this study may further increase due to the reduced salivary clearance rate that enhances the medications’ components and pigments to be absorbed by the restorative materials [21,24,35,38].

The present study has certain limitations as it could not completely simulate the oral environment. The results may vary due to the neutralizing effect of saliva, thermal changes and microorganisms present in the oral cavity. To overcome the limitations of this study, laboratory studies that add artificial saliva in the assessment models and clinical studies of the dental structures and restorative materials after anti-asthmatic medication administration and brushing are needed.

## 5. Conclusions

The present study showed the following:

- Salbutamol and Budesonide significantly decreased the microhardness of enamel and flowable composite samples after 10 days of administration and brushing.
- After 10 days of Salbutamol or Budesonide administration and brushing, flowable composite showed lower microhardness and lower surface roughness.
- Packable composite showed more resistance to microhardness change.
- Longer application of any of the medications resulted in lowering the surface roughness of both flowable and packable composites. Budesonide showed more reduction in surface roughness than Salbutamol after 10 days.
- Both medications showed a clinically detectable change in color of the packable and flowable composites, with Budesonide showing a greater change than Salbutamol after 10 days.
- Despite the improvements in composite resin materials, clinicians should consider the long-term durability of restorative materials.

## Figures and Tables

**Table 1:** Restorative composite material composition.

Composite Type	Manufacture	Composition
Nanohybrid packable composite	Tetric^®^ N-Ceram, TBF, Ivoclar/Vivadent	Dimethacrylates (19–20 wt.%) The fillers are barium glass, ytterbium trifluoride, mixed oxide and copolymers (80–81 wt.%)
Nanofilled flowable composite	Tetric^®^ N-Flow, TBF, Ivoclar/Vivadent	Monomethacrylates and dimethacrylates (28 wt%) The fillers are barium glass, ytterbium trifluoride and copolymers (71 wt%). Additives, initiators, stabilizers and pigments are additional ingredients (<1.0 wt%).

**Table 2:** Medications used and their doses.

Group	Inhaler Type	Composition	Tested Dose
1	Salbutamol (Ventolin Nebules, Glaxo Smith Kline, Boronia, Vic., Australia) (100 mcg/actuation)	Salbutamol, sodium chloride, sulfuric acid, distilled water	0.6 mL/6 mL saline
2	Budesonide, Pulmicort^®^ Nebulising Suspension (0.5 mcg/actuation)	Budesonide, disodium edetate, sodium chloride, polysorbate, citric acid, monohydrate, sodium citrate, distilled water	2 mL/2 mL saline

**Table 3:** Means and standard deviations for microhardness.

Medication	Group	Microhardness (KH for Enamel or VH for Composite)		
		T_0_	T_1_	T_2_
Salbutamol	Enamel samples	498.98 ± 24.25 ^a^	120.43 ± 59 ^b^	92.86 ± 54.40 ^b^
	Packable composite	95.16 ± 22.01 ^a^	84.73 ± 12.87 ^ab^	64.67 ± 14.12 ^b^
	Flowable composite	71.25 ± 13.37 ^a^	62.18 ± 10.62 ^a^	43.28 ± 9.44 ^b^
Budesonide	Enamel samples	420.90.57 ± 34.80 ^a^	169.65 ± 79.62 ^b^	127.15 ± 84.30 ^b^
	Packable composite	89.26 ± 18.03 ^a^	75.35 ± 12.55 ^ab^	69.91 ± 12.63 ^b^
	Flowable composite	76.41 ± 13.97 ^a^	52.82 ± 10.54 ^b^	51.95 ± 11.82 ^b^

Note: Values with different superscript small letters in the same row are statistically significantly different (*p* < 0.05). There were no significant differences in microhardness between the two medications in the same material’s group and time interval (*p* > 0.05).

**Table 4:** Means and standard deviations for surface roughness.

Medication	Group	Surface Roughness (nm)		
		T_0_	T_1_	T_2_
Salbutamol	Packable composite	714.94 ± 42.34 ^a^	632.89 ± 84.58 ^ab^	585.93 ± 112.90 ^bA^
	Flowable composite	686.47 ± 95.95 ^a^	535.74 ± 114.32 ^b^	500.71 ± 110.95 ^bA^
Budesonide	Packable composite	700.89 ± 119.35 ^a^	705.33 ± 135.59 ^a^	335.32 ± 57.58 ^bB^
	Flowable composite	649.28 ± 50.39 ^a^	519.34 ± 91.08 ^b^	372.22 ± 97.20 ^cB^

Note: Values with different superscript small letters in the same row are statistically significantly different (*p* < 0.05). Different superscript capital letters are used only when there is a significant difference between the effects of the two medications on the same material at the same time points (*p* < 0.05).

**Table 5:** Means and standard deviations for color change.

Medication	Group	Color Change (ΔE*)	
		T_1_	T_2_
Salbutamol	Packable composite	4.99 ± 1.68	5.32 ± 1.30
	Flowable composite	4.59 ± 1.63	4.68 ± 2.00 ^A^
Budesonide	Packable composite	4.23 ± 2.17	4.23 ± 1.76
	Flowable composite	6.02 ± 1.98	6.94 ± 1.42 ^B^

Note: Different superscript capital letters are used only when there is a significant difference between the effects of the two medications on the same material at the same time points (*p* < 0.05). There were no significant differences in color change between the two time intervals in the same material’s group and medication (*p* > 0.05).

## Data Availability

The data presented in this study are available on request from the corresponding author.
